# The association between polymorphisms in microRNA genes and cervical cancer in a Chinese Han population

**DOI:** 10.18632/oncotarget.21235

**Published:** 2017-09-23

**Authors:** Li Chuanyin, Wang Xiaona, Yan Zhiling, Zhang Yu, Liu Shuyuan, Yang Jie, Hong Chao, Shi Li, Yang Hongying, Yao Yufeng

**Affiliations:** ^1^ Institute of Medical Biology, Chinese Academy of Medical Sciences & Peking Union Medical College, Kunming 650118, China; ^2^ Department of Gynaecologic Oncology, The 3rd Affiliated Hospital of Kunming Medical University, Kunming 650118, China; ^3^ Wenzhou Medical University, Wenzhou 325035, China

**Keywords:** association study, cervical cancer, microRNA, single nucleotide polymorphisms, Chinese Han population

## Abstract

Several studies have confirmed the crucial roles of microRNAs (miRNAs) in cancer occurrence. In addition, single nucleotide polymorphisms (SNPs) in miRNA genes have been associated with various cancers. The aim of the present study was to investigate the association of SNPs in miRNA genes with cervical intraepithelial neoplasia (CIN) and cervical cancer in a Chinese Han population. We searched SNPs in nineteen miRNAs by sequencing healthy individuals (n=50). Then, a total of 400 patients with CIN, 609 patients with cervical cancer and 583 healthy individuals were recruited to genotype the SNPs using a Taqman assay. The results showed that only five of the nineteen miRNAs had SNPs (rs11134527 in pri-miR-218-2; rs74693964 in pri-miR-145; rs6062251 in pri-miR-133a2; rs531564 in pri-miR-124-1; and rs1834306 in pri-miR-100) in this Chinese Han population. The frequency of the rs11134527A allele was significantly higher in the control group than in CIN and cervical cancer groups (*P*=0.011 and 0.035, respectively). The frequency of the rs531564G allele was higher in the CIN and control groups than in the cervical cancer group (*P*=0.019 and 0.017, respectively). These results indicated that rs11134527 in pri-miR-218-2 and rs531564 in pri-miR-124-1 could be associated with CIN and cervical cancer in the Chinese Han population.

## INTRODUCTION

Cervical cancer is one of the most common malignancies in women worldwide, with an estimated global incidence of 470,000 new cases and approximately 233,000 deaths per year [1]. Compared to developed countries, the incidence and mortality of cervical cancer is higher in low-resource countries [2]. High-risk human papillomavirus (HPV) infections are the main aetiological factors of cervical intraepithelial neoplasia (CIN) in women, and persisting high-risk HPV infections significantly facilitate the development of CIN to cervical cancer. However, evidence suggests that host genetic factors are also involved in the initiation and development of cervical cancer [3–5].

MicroRNAs (miRNAs) are a group of short RNAs with 20-23 noncoding nucleotides. MiRNAs play important roles in various biological processes through binding to the 3′-untranslate region (UTR) of target mRNAs and inhibiting protein translation or degrading mRNAs. MiRNAs regulate more than 60% of all human genes [6] and are involved in crucial physiological processes, such as proliferation, apoptosis, differentiation, tumourigenesis and cancer metastasis [7–10].

Recent studies have demonstrated the aberrant expression of miRNAs in cervical cancer and their contribution to tumour generation [11]. Functional studies have shown that miRNAs impact tumour cell fate through the regulation of various cell signalling pathways [12]. Thus, miRNAs act as oncogenic or anti-oncogenic molecules in the initiation and development of cervical cancer [13]. For example, miR-218 functions as a tumour suppressor through targeting focal adhesion pathways and the SLIT2-ROBO1 pathway, and inhibiting cancer cell migration and invasion. Single nucleotide polymorphisms (SNPs) in miRNA genes have been associated with various human diseases [14–16]. In 2016, Wang *et al*. reported that rs767649 in miR-155 is associated with cervical cancer [17].

In the present study, we searched for SNPs in nineteen miRNAs associated with cervical cancer [18]. Subsequently, we conducted an association study in patients with CIN, patients with cervical cancer and healthy individuals to investigate the relationship of the SNPs in miRNAs with CIN and cervical cancer in a Chinese Han population.

## RESULTS

### Subject characteristics

Table 1 lists the characteristics of subjects in the present study. There was no significant difference in age between CIN, cervical cancer and control groups (*p*>0.05). In the cervical cancer group, there were 91 individuals with adenocarcinoma (AC), 501 individuals with squamous cell carcinoma (SCC) and 17 individuals with other pathological types.

**Table 1:** The characteristics of the subjects in the present subjects

	Cervical cancer	CIN	Control	*P* value
N	609	400	583	
Ages	44.22±9.08	43.46±9.25	43.89±5.93	0.345
**Pathologic types**				
SCC	501			
AC	91			
Others	17			

### The sequencing results of nineteen miRNA genes

Five of the nineteen miRNAs showed polymorphisms in 50 healthy individuals. The polymorphisms are rs11134527 in pri-miR-218-2, rs74693964 in pri-miR-145, rs6062251 in pri-miR-133a2, rs531564 in pri-miR-124-1 and rs1834306 in pri-miR-100.

### Association of the five SNPs with cervical cancer

The allelic and genotypic frequencies for the five SNPs in miRNAs are displayed in Tables 2 and 3, respectively. All five SNPs were in Hardy-Weinberg equilibrium (HWE) in the control group (*P*>0.05). The A allelic frequency of rs11134527 in CIN (59.3%) and cervical cancer (60.8%) groups was significantly different from that in the control group(64.9%) (*P*=0.011 and 0.035, respectively), and the A allele occurred more frequently in the control group compared with the CIN (OR=0.786; 95%CI: 0.653-0.946) and cervical cancer groups (OR=0.836; 95%CI: 0.708-0.988). The C allelic frequency of rs531564 in the cervical cancer group (14.6%) was significantly different from that in the control (11.3%) and CIN groups (11.0%) (*P*=0.017 and 0.019, respectively), and the C allele occurred more frequently in the cervical cancer group compared to the control groups (OR=1.341;95%CI:1.054-1.706) and CIN (OR=1.385;95%CI:1.054-1.819). The genotypic frequencies of rs11134527 (A/A, 36.7%; A/G, 45.0%; and G/G, 18.2%) and rs6062251 (C/C, 8.5%; C/T, 51.7%; and T/T, 39.8%) in the CIN group were significantly different from those in the control (A/A, 41.5%; A/G, 46.8%; and G/G, 11.7%) (*P*=0.013) and cervical cancer groups (C/C, 12.8%; C/T, 42.5%; and T/T, 44.7%) (*P*=0.007), respectively. Moreover, the genotypic frequencies of rs531564 in the cervical cancer group (C/C,2.8%; C/G,23.6%; and G/G,73.6%) were significantly different from that in the CIN (C/C,1.0%; C/G,20.0%; and G/G,79.0%) and control groups (C/C,1.2%; C/G,20.2%; and G/G,78.6%) (*P*=0.047 and 0.043, respectively). The allelic and genotypic frequencies of rs74693964 and rs1834306 were not significantly different among the three groups.

**Table 2:** Allelic frequencies comparison of the five SNPs among control, CIN and cervical cancer groups

SNPs	Alleles(*n*, %)		Control VS CIN		CIN VS cervical cancer		Control VS cervical cancer	
			*P* value	OR[95%CI]	*P* value	OR[95%CI]	*P* value	OR[95%CI]
rs11134527	A	G	**0.011**	**0.786[0.653-0.946]**	0.499	1.065[0.888-1.277]	**0.035**	**0.836[0.708-0.988]**
Control	757(64.9%)	409(35.1%)						
CIN	474(59.3%)	326(40.7%)						
Cervical cancer	740(60.8%)	478(39.2%)						
rs74693964	C	T	0.502	1.187[0.719-1.960]	0.585	1.158[0.684-1.963]	0.174	1.375[0.867-2.180]
Control	1123(96.3%)	43(3.7%)						
CIN	775(96.9%)	25(3.1%)						
Cervical cancer	1185(97.3%)	33(2.7%)						
rs6062251	C	T	0.912	1.011[0.836-1.222]	0.888	0.987[0.818-1.191]	0.975	0.997[0.842-1.181]
Control	398(34.1%)	768(65.9%)						
CIN	275(34.4%)	525(65.6%)						
Cervical cancer	415(34.1%)	803(65.9%)						
rs531564	C	G	0.825	0.968[0.727-1.289]	**0.019**	**1.385[1.054-1.819]**	**0.017**	**1.341[1.054-1.706]**
Control	132(11.3%)	1034(88.7%)						
CIN	88(11.0%)	712(89.0%)						
Cervical cancer	178(14.6%)	1040(85.4%)						
rs1834306	A	G	0.964	1.004[0.838-1.203]	0.701	1.036[0.866-1.238]	0.634	1.040[0.885-1.221]
Control	541(46.4%)	625(53.6%)						
CIN	372(46.5%)	428(53.5%)						
Cervical cancer	577(47.4%)	641(52.6%)						

**Table 3:** Genotypic frequencies comparison of the five SNPs among control, CIN and cervical cancer groups

SNPs	Genotypes(*n*, %)			*P* value		
				Control VS CIN	CIN VS cervical cancer	Control VS cervical cancer
rs11134527	A/A	A/G	G/G	**0.013**	0.366	0.101
Control	242(41.5%)	273(46.8%)	68(11.7%)			
CIN	147(36.7%)	180(45.0%)	73(18.2%)			
Cervical cancer	233(36.6%)	294(48.3%)	92(15.1%)			
rs74693964	C/C	C/T	T/T	0.702	0.454	0.302
Control	541(92.8%)	41(7.0%)	1(0.2%)			
CIN	376(94.0%)	23(5.8%)	1(0.3%)			
Cervical cancer	576(94.6%)	33(5.4%)	0(0.0%)			
rs6062251	C/C	C/T	T/T	0.225	**0.007**	0.293
Control	63(10.8%)	272(46.7%)	248(42.5%)			
CIN	34(8.5%)	207(51.7%)	159(39.8%)			
Cervical cancer	78(12.8%)	259(42.5%)	272(44.7%)			
rs531564	C/C	C/G	G/G	**0.952**	**0.047**	**0.043**
Control	7(1.2%)	118(20.2%)	458(78.6%)			
CIN	4(1.0%)	80(20.0%)	316(79.0%)			
Cervical cancer	17(2.8%)	144(23.6%)	448(73.6%)			
rs1834306	A/A	A/G	G/G	0.811	0.834	0.875
Control	126(21.6%)	289(49.6%)	168(28.8%)			
CIN	91(22.8%)	190(47.5%)	119(29.7%)			
Cervical cancer	139(22.8%)	299(49.1%)	171(28.1%)			

### Model of inheritance analysis

Tables 4, 5, 6 present the results of the analyses of different models of inheritance for the five SNPs. The Akaike information criterion (AIC) and Bayesian information criterion (BIC) were calculated to identify the best inheritance model for each SNP. The model with smallest AIC and BIC values was the best-fit inheritance model. The genotypic frequency of rs11134527 A/A-A/G was significantly different from G/G (*P*=0.018) under the recessive model between control and CIN groups. In this model, the A/A-A/G genotype was associated with a decreased risk of CIN (OR=0.61; 95%CI: 0.41-0.92). A comparison between CIN and cervical cancer groups revealed that the genotypic frequencies of rs6062251 were significantly different in the overdominant model (*P*=0.007). In the overdominant model, the T/T-C/C genotype of rs6062251 was associated with increased risk of cervical cancer (OR=1.45; 95%CI:1.11-1.90). A comparison of control and cervical cancer groups revealed that the genotypic frequencies of rs531564 were significantly different in the log-additive model (*P*=0.022). In this model, the genotype 2G/G-C/G of rs531564 (OR=0.75; 95%CI: 0.58-0.96) was associated with a decreased risk of cervical cancer.

**Table 4:** Different inheritance models analysis of the SNPs in miRNA genes in the comparison between control and CIN groups

SNPs	Model	Genotypes	CIN (*n*, %)	Control (*n*, %)	After adjusted by Age			
					OR (95% CI)	*P* value	AIC	BIC
rs11134527
	Codominant	A/A	147 (36.8%)	242 (41.5%)	1	0.062	1215.4	1450.2
		A/G	180 (45.0%)	273 (46.8%)	0.99 (0.72-1.35)			
		G/G	73 (18.2%)	68 (11.7%)	0.61 (0.39-0.94)			
	Dominant	A/A	147 (36.8%)	242 (41.5%)	1	0.390	1218.3	1448.1
		A/G-G/G	253 (63.2%)	341 (58.5%)	0.88 (0.66-1.18)			
	Recessive	A/A-A/G	327 (81.8%)	515 (88.3%)	**1**	**0.018**	**1213.4**	**1443.3**
		G/G	73 (18.2%)	68 (11.7%)	**0.61 (0.41-0.92)**			
	Overdominant	A/A-G/G	220 (55.0%)	310 (53.2%)	1	0.420	1218.3	1448.2
		A/G	180 (45.0%)	273 (46.8%)	1.13 (0.85-1.50)			
	Log-additive	---	---	---	0.82 (0.67-1.02)	0.069	1215.7	1445.5
rs74693964
	Codominant	C/C	376 (94%)	541 (92.8%)	1	0.590	1220.0	1454.7
		C/T	23 (5.8%)	41 (7.0%)	1.37 (0.74-2.54)			
		T/T	1 (0.2%)	1 (0.2%)	1.34 (0.05-32.99)			
	Dominant	C/C	376 (94.0%)	541 (92.8%)	1	0.310	1218.0	1447.8
		C/T-T/T	24 (6.0%)	42 (7.2%)	1.37 (0.74-2.52)			
	Recessive	C/C-C/T	399 (99.8%)	582 (99.8%)	1	0.870	1219.0	1448.8
		T/T	1 (0.2%)	1 (0.2%)	1.32 (0.05-32.33)			
	Overdominant	C/C-T/T	377 (94.2%)	542 (93.0%)	1	0.310	1218.0	1447.8
		C/T	23 (5.8%)	41 (7.0%)	1.37 (0.74-2.54)			
	Log-additive	---	---	---	1.34 (0.75-2.39)	0.310	1218.0	1447.9
rs6062251
	Codominant	T/T	159 (39.8%)	248 (42.5%)	1	0.200	1217.7	1452.5
		C/T	207 (51.8%)	272 (46.7%)	0.78 (0.58-1.06)			
		C/C	34 (8.5%)	63 (10.8%)	1.07 (0.64-1.80)			
	Dominant	T/T	159 (39.8%)	248 (42.5%)	1	0.180	1217.2	1447.1
		C/T-C/C	241 (60.2%)	335 (57.5%)	0.82 (0.61-1.10)			
	Recessive	T/T-C/T	366 (91.5%)	520 (89.2%)	1	0.410	1218.3	1448.2
		C/C	34 (8.5%)	63 (10.8%)	1.23 (0.75-2.00)			
	Overdominant	T/T-C/C	193 (48.2%)	311 (53.3%)	1	0.074	1215.8	1445.7
		C/T	207 (51.8%)	272 (46.7%)	0.77 (0.58-1.03)			
	Log-additive	---	---	---	0.93 (0.74-1.16)	0.520	1218.6	1448.5
rs531564
	Codominant	G/G	316 (79.0%)	458 (78.6%)	1	0.860	1220.7	1455.5
		C/G	80 (20.0%)	118 (20.2%)	0.96 (0.67-1.36)			
		C/C	4 (1.0%)	7 (1.2%)	1.39 (0.35-5.57)			
	Dominant	G/G	316 (79.0%)	458 (78.6%)	1	0.880	1219.0	1448.8
		C/G-C/C	84 (21.0%)	125 (21.4%)	0.97 (0.69-1.38)			
	Recessive	G/G-C/G	396 (99.0%)	576 (98.8%)	1	0.630	1218.8	1448.6
		C/C	4 (1.0%)	7 (1.2%)	1.40 (0.35-5.62)			
	Overdominant	G/G-C/C	320 (80.0%)	465 (79.8%)	1	0.780	1218.9	1448.8
		C/G	80 (20.0%)	118 (20.2%)	0.95 (0.67-1.36)			
	Log-additive	---	---	---	1.00 (0.72-1.38)	0.980	1219.0	1448.9
rs1834306
	Codominant	G/G	119 (29.8%)	168 (28.8%)	1	0.750	1220.4	1455.2
		A/G	190 (47.5%)	289 (49.6%)	1.13 (0.81-1.58)			
		A/A	91 (22.8%)	126 (21.6%)	1.05 (0.70-1.56)			
	Dominant	G/G	119 (29.8%)	168 (28.8%)	1	0.530	1218.6	1448.5
		A/G-A/A	281 (70.2%)	415 (71.2%)	1.10 (0.81-1.51)			
	Recessive	G/G-A/G	309 (77.2%)	457 (78.4%)	1	0.860	1219.0	1448.8
		A/A	91 (22.8%)	126 (21.6%)	0.97 (0.69-1.37)			
	Overdominant	G/G-A/A	210 (52.5%)	294 (50.4%)	1	0.470	1218.5	1448.3
		A/G	190 (47.5%)	289 (49.6%)	1.11 (0.84-1.48)			
	Log-additive	---	---	---	1.03 (0.84-1.26)	0.770	1218.9	1448.8

**Table 5:** Different inheritance models analysis of the SNPs in miRNA genes in the comparison between CIN and cervical cancer groups

SNPs	Models	Genotypes	Cervical cancer (*n*, %)	CIN (*n*, %)	After adjusted by Age			
					OR (95% CI)	*P* value	AIC	BIC
rs11134527
	Codominant	A/A	223 (36.6%)	147 (36.8%)	1	0.290	1365.1	1615.8
		A/G	294 (48.3%)	180 (45.0%)	0.89 (0.66-1.19)			
		G/G	92 (15.1%)	73 (18.2%)	1.20 (0.81-1.78)			
	Dominant	A/A	223 (36.6%)	147 (36.8%)	1	0.770	1365.5	1611.3
		A/G-G/G	386 (63.4%)	253 (63.2%)	0.96 (0.73-1.27)			
	Recessive	A/A-A/G	517 (84.9%)	327 (81.8%)	1	0.170	1363.7	1609.5
		G/G	92 (15.1%)	73 (18.2%)	1.28 (0.90-1.84)			
	Overdominant	A/A-G/G	315 (51.7%)	220 (55.0%)	1	0.200	1363.9	1609.7
		A/G	294 (48.3%)	180 (45.0%)	0.84 (0.64-1.10)			
	Log-additive	---	---	---	1.05 (0.87-1.27)	0.600	1365.3	1611.1
rs74693964
	Codominant	C/C	576 (94.6%)	376 (94.0%)	1	0.510	1366.2	1617
		C/T	33 (5.4%)	23 (5.8%)	0.96 (0.54-1.73)			
		T/T	0 (0.0%)	1 (0.2%)	NA (0.00-NA)			
	Dominant	C/C	576 (94.6%)	376 (94.0%)	1	1	1365.5	1611.4
		C/T-T/T	33 (5.4%)	24 (6.0%)	1.00 (0.56-1.78)			
	Recessive	C/C-C/T	609 (100.0%)	399 (99.8%)	1	0.250	1364.2	1610.1
		T/T	0 (0.0%)	1 (0.2%)	NA (0.00-NA)			
	Overdominant	C/C-T/T	576 (94.6%)	377 (94.2%)	1	0.890	1365.5	1611.4
		C/T	33 (5.4%)	23 (5.8%)	0.96 (0.54-1.72)			
	Log-additive	---	---	---	1.04 (0.60-1.82)	0.890	1365.5	1611.4
rs6062251
	Codominant	T/T	272 (44.7%)	159 (39.8%)	1	0.017	1359.4	1610.2
		C/T	259 (42.5%)	207 (51.8%)	1.39 (1.05-1.85)			
		C/C	78 (12.8%)	34 (8.5%)	0.81 (0.50-1.31)			
	Dominant	T/T	272 (44.7%)	159 (39.8%)	1	0.088	1362.6	1608.5
		C/T-C/C	337 (55.3%)	241 (60.2%)	1.27 (0.96-1.67)			
	Recessive	T/T-C/T	531 (87.2%)	366 (91.5%)	1	0.089	1362.7	1608.5
		C/C	78 (12.8%)	34 (8.5%)	0.68 (0.43-1.07)			
	Overdominant	T/T-C/C	350 (57.5%)	193 (48.2%)	**1**	**0.007**	**1358.1**	**1604.0**
		C/T	259 (42.5%)	207 (51.8%)	**1.45 (1.11-1.90)**			
	Log-additive	---	---	---	1.05 (0.86-1.29)	0.620	1365.3	1611.1
rs531564
	Codominant	G/G	448 (73.6%)	316 (79.0%)	1	0.072	1362.3	1613.0
		C/G	144 (23.6%)	80 (20.0%)	0.85 (0.61-1.18)			
		C/C	17 (2.8%)	4 (1.0%)	0.32 (0.11-1.00)			
	Dominant	G/G	448 (73.6%)	316 (79.0%)	1	0.140	1363.3	1609.2
		C/G-C/C	161 (26.4%)	84 (21.0%)	0.79 (0.58-1.08)			
	Recessive	G/G-C/G	592 (97.2%)	396 (99.0%)	1	0.038	1361.2	1607.1
		C/C	17 (2.8%)	4 (1.0%)	0.34 (0.11-1.03)			
	Overdominant	G/G-C/C	465 (76.3%)	320 (80.0%)	1	0.400	1364.8	1610.7
		C/G	144 (23.6%)	80 (20.0%)	0.87 (0.63-1.20)			
	Log-additive	---	---	---	0.76 (0.58-1.01)	0.057	1361.9	1607.8
rs1834306
	Codominant	G/G	171 (28.1%)	119 (29.8%)	1	0.84	1367.2	1617.9
		A/G	299 (49.1%)	190 (47.5%)	0.92 (0.67-1.26)			
		A/A	139 (22.8%)	91 (22.8%)	0.90 (0.62-1.32)			
	Dominant	G/G	171 (28.1%)	119 (29.8%)	1	0.56	1365.2	1611
		A/G-A/A	438 (71.9%)	281 (70.2%)	0.92 (0.68-1.23)			
	Recessive	G/G-A/G	470 (77.2%)	309 (77.2%)	1	0.75	1365.4	1611.3
		A/A	139 (22.8%)	91 (22.8%)	0.95 (0.69-1.31)			
	Overdominant	G/G-A/A	310 (50.9%)	210 (52.5%)	1	0.79	1365.5	1611.3
		A/G	299 (49.1%)	190 (47.5%)	0.96 (0.74-1.26)			
	Log-additive	---	---	---	0.95 (0.78-1.14)	0.58	1365.2	1611.1

**Table 6:** Different inheritance models analysis of the SNPs in miRNA genes in the comparison between control and cervical cancer groups

SNPs	Model	Genotypes	Cervical cancer (*n*, %)	Control (*n*, %)	After adjusted by Age			
					OR (95% CI)	*P* value	AIC	BIC
rs11134527
	Codominant	A/A	223 (36.6%)	242 (41.5%)	1	0.190	1577.2	1826.3
		A/G	294 (48.3%)	273 (46.8%)	0.83 (0.64-1.08)			
		G/G	92 (15.1%)	68 (11.7%)	0.72 (0.49-1.07)			
	Dominant	A/A	223 (36.6%)	242 (41.5%)	1	0.095	1575.7	1819.7
		A/G-G/G	386 (63.4%)	341 (58.5%)	0.81 (0.63-1.04)			
	Recessive	A/A-A/G	517 (84.9%)	515 (88.3%)	1	0.230	1577.1	1821.1
		G/G	92 (15.1%)	68 (11.7%)	0.80 (0.56-1.15)			
	Overdominant	A/A-G/G	315 (51.7%)	310 (53.2%)	1	0.420	1577.9	1821.9
		A/G	294 (48.3%)	273 (46.8%)	0.90 (0.71-1.15)			
	Log-additive	---	---	---	0.85 (0.70-1.01)	0.070	1575.2	1819.2
rs74693964
	Codominant	C/C	576 (94.6%)	541 (92.8%)	1	0.440	1578.9	1828.0
		C/T	33 (5.4%)	41 (7.0%)	1.23 (0.75-2.04)			
		T/T	0 (0.0%)	1 (0.2%)	NA (0.00-NA)			
	Dominant	C/C	576 (94.6%)	541 (92.8%)	1	0.370	1577.7	1821.7
		C/T-T/T	33 (5.4%)	42 (7.2%)	1.26 (0.76-2.07)			
	Recessive	C/C-C/T	609 (100.0%)	582 (99.8%)	1	0.320	1577.6	1821.6
		T/T	0 (0.0%)	1 (0.2%)	NA (0.00-NA)			
	Overdominant	C/C-T/T	576 (94.6%)	542 (93%)	1	0.420	1577.9	1821.9
		C/T	33 (5.4%)	41 (7.0%)	1.23 (0.74-2.03)			
	Log-additive	---	---	---	1.28 (0.78-2.09)	0.330	1577.6	1821.6
rs6062251
	Codominant	T/T	272 (44.7%)	248 (42.5%)	1	0.480	1579.1	1828.2
		C/T	259 (42.5%)	272 (46.7%)	1.12 (0.86-1.45)			
		C/C	78 (12.8%)	63 (10.8%)	0.90 (0.60-1.34)			
	Dominant	T/T	272 (44.7%)	248 (42.5%)	1	0.600	1578.2	1822.2
		C/T-C/C	337 (55.3%)	335 (57.5%)	1.07 (0.83-1.37)			
	Recessive	T/T-C/T	531 (87.2%)	520 (89.2%)	1	0.390	1577.8	1821.8
		C/C	78 (12.8%)	63 (10.8%)	0.85 (0.58-1.24)			
	Overdominant	T/T-C/C	350 (57.5%)	311 (53.3%)	1	0.280	1577.4	1821.4
		C/T	259 (42.5%)	272 (46.7%)	1.14 (0.90-1.46)			
	Log-additive	---	---	---	1.00 (0.83-1.20)	0.980	1578.5	1822.5
rs531564
	Codominant	G/G	448 (73.6%)	458 (78.6%)	1	0.038	1574.0	1823.1
		C/G	144 (23.6%)	118 (20.2%)	0.81 (0.61-1.10)			
		C/C	17 (2.8%)	7 (1.2%)	0.36 (0.15-0.91)			
	Dominant	G/G	448 (73.6%)	458 (78.6%)	1	0.060	1575.0	1819
		C/G-C/C	161 (26.4%)	125 (21.4%)	0.76 (0.57-1.01)			
	Recessive	G/G-C/G	592 (97.2%)	576 (98.8%)	1	0.031	1573.9	1817.9
		C/C	17 (2.8%)	7 (1.2%)	0.38 (0.15-0.95)			
	Overdominant	G/G-C/C	465 (76.3%)	465 (79.8%)	1	0.23	1577.1	1821.1
		C/G	144 (23.6%)	118 (20.2%)	0.84 (0.62-1.12)			
	Log-additive	**---**	**---**	**---**	**0.75 (0.58-0.96)**	**0.022**	**1573.3**	**1817.3**
rs1834306
	Codominant	G/G	171 (28.1%)	168 (28.8%)	1	1	1580.5	1829.6
		A/G	299 (49.1%)	289 (49.6%)	0.99 (0.74-1.32)			
		A/A	139 (22.8%)	126 (21.6%)	0.99 (0.70-1.41)			
	Dominant	G/G	171 (28.1%)	168 (28.8%)	1	0.93	1578.5	1822.5
		A/G-A/A	438 (71.9%)	415 (71.2%)	0.99 (0.75-1.30)			
	Recessive	G/G-A/G	470 (77.2%)	457 (78.4%)	1	0.99	1578.5	1822.5
		A/A	139 (22.8%)	126 (21.6%)	1.00 (0.74-1.35)			
	Overdominant	G/G-A/A	310 (50.9%)	294 (50.4%)	1	0.93	1578.5	1822.5
		A/G	299 (49.1%)	289 (49.6%)	0.99 (0.77-1.26)			
	Log-additive	---	---	---	1.00 (0.84-1.19)	0.96	1578.5	1822.5

## DISCUSSION

Evidence has shown that miRNAs are involved in the initiation and development of various types of cancers, and these molecules function as anti-oncogenes or oncogenes. SNPs in miRNAs, including primary, precursor and mature miRNA, may change the expression level and alter the binding affinity of miRNAs, and further regulate the expression of the target genes, thereby markedly impacting cell pathway regulation, which defines cellular fates [19, 20]. In the present study, we observed that rs11134527 in pri-miR-218-2 and rs531564 in pri-miR-124-1 were associated with CIN and cervical cancer in a Chinese Han population.

In 2013, Yamamoto N *et al*. reported that the expression of miR-218 was significantly lower in cervical cancer tissues than in adjacent non-cancerous tissues, and the restoration of miR-218 significantly inhibited cancer cell proliferation, migration and invasion [21]. In addition, the anti-oncogene function of miR-218 has been reported in several types of cancers through targeting several oncogenes, such as Rictor (oral cancer), EGFR (non-small cell lung cancer), ROBO1 receptor (gastric cancer) and LAMB3 (cervical cancer) [22–25]. Several studies have investigated the association of rs11134527 with different diseases (Table 7) [26–28]; however, these studies provided contradictory results. In the present study, rs11134527 in pri-miR-218-2 was associated with CIN and cervical cancer in a Chinese Han population, and the A allele of rs11134527 was associated with a lower risk of CIN (*P*=0.011; OR=0.786; 95%CI:0.653-0.946) and cervical cancer (*P*=0.035; OR=0.836; 95%CI:0.708-0.988) in a comparison of CIN and cervical cancer groups with control group. The genotype A/A-A/G showed a lower risk of cervical cancer compared with the G/G genotype using the recessive model in a comparison of the CIN group with the control group (*P*=0.018; OR=0.61;95%CI:0.41-0.92). However, this result was inconsistent with Shi *et al*. [28] whose study showed that the G/G genotype was associated with a decreased risk of cervical cancer compared with AA and A/G genotypes. The discrepancy between these results might reflect different sample sizes and populations in the two studies, and unfortunately, the Shi *et al*. study did not focus on the association with CIN, thus, we could not compare the results between CIN and the control. In the present study, the allelic frequencies of rs11134527 were only significantly different in the comparison of CIN and cervical cancer groups with control group, but not in the comparison of CIN group with cervical cancer group.

**Table 7:** The characteristics of five SNPs found in miRNAs and their related association studies with cancers

miRNA locus	Function	SNPs	Alleles (case/control)		Diseases/reference	Population(region)	*P* value
Pri-miR-218-2	Anti-oncogene	rs11134527	A	G			
			1321/1538	897/1012	ESCC [26]	Han Chinese (Taizhou and Shanghai)	0.595
			346/599	258/427	HCC [27]	Han Chinese (Sichuan)	0.670
			1928/1662	1202/1120	Cervical Cancer [28]	Han Chinese (Shanghai)	0.145
Pri-miR-124-1	Anti-oncogene	rs531564	C	G			
			1901/2151	317/399	ESCC [26]	Han Chinese (Taizhou and Shanghai)	0.191
			290/434	26/86	Cervical Cancer [37]	Han Chinese (Zhengzhou)	0.001
			197/353	17/63	Cervical Cancer [38]	Han Chinese (Guangdong)	0.014
Pri-miR-145	Anti-oncogene	rs74693964	C	T			
			no reference	no reference	no reference	no reference	no reference
Pri-miR-133a2	Anti-oncogene	rs6062251	C	T			
			no reference	no reference	no reference	no reference	no reference
Pri-miR-100	Anti-oncogene	rs1834306	A	G			
			217/189	279/399	ESCC [50]	Kazakh Chinese (Xinjiang)	8.37×10^−5^
			92/377	114/457	Cervical Cancer [51]	Han Chinese (Guangzhou)	0.892

Recent studies have reported that miR-124 suppresses a series of genes involved in the growth of human cancers. MiR-124 suppresses tumour growth through targeting STAT3 in colorectal cancer [29], CD151 in breast cancer [30], and ERK in cutaneous squamous cell carcinoma [31]. In cervical cancer, Wan *et al*. reported that miR-124 represses vasculogenic mimicry and cell motility by targeting amotL1 [32], and Zhang *et al*. showed that miR-124 inhibits the proliferation, invasion, migration and epithelial-mesenchymal transition of cervical cancer [33]. As an anti-oncogene, significantly lower miR-124 expression was detected in cervical cancer than in low-grade squamous intraepithelial lesions [34]. Studies have shown that SNPs in miRNA genes might alter miRNA expression or function [35, 36]. Thus, SNPs in miR-124 might be associated with miR-124 expression and are further associated with cervical cancer. Rs531564 has been significantly correlated with the risk of various cancers (Table 7), including cervical cancer and oesophageal squamous cell carcinoma (ESCC) [26, 37, 38]. In the present study, rs531564 in pri-miR-124-1 was associated with cervical cancer risk (*P*=0.017, OR=1.341; 95%CI: 1.054-1.706). Additionally, this SNP was correlated with the progression from CIN to cervical cancer (*P*=0.019; OR=1.385; 95%CI:1.054~1.819). These results were consistent with the results of Wu *et al*., showing that the C allele is a risk factor for cervical cancer [37]. However, there was no significant association of rs531564 with CIN in the present study. It is likely that this polymorphism in pri-miR-124-1 is only associated with cervical cancer but not CIN. The two different stages (CIN and cervical cancer) of cervical cancer have different nosogenesis; thus, the same SNP may play different roles in two different disease stages.

MiR-145, a tumour suppressor, has been implicated in the death-promoting regulatory loop of P53 [39]. The downregulation of miR-145 has been associated with aggressive progression and poor prognosis in cervical cancer [40]. In 2015, Chacon-Cortes *et al* reported the association of rs353291 in miR-145 with breast cancer susceptibility [41]. However, in the present study, we investigated the association of rs74693964, another SNP in near Gene-3 of miR-145, with cervical cancer in a Chinese population; no significant association of rs74693964 with CIN and cervical cancer was detected.

MiR-133a plays a crucial role in myoblast proliferation and differentiation. Recent studies have shown the downregulation of miR-133a in human cancers. Accumulating evidence has indicated that the restoration of miR-133a expression could induce the suppression of cell proliferation, migration and invasion, and tumour cell apoptosis [42–44], indicating that miR-133a might act as a tumour suppressor in human cancers, including cervical cancer [45]. Zhou *et al* reported that rs8089787 and rs9948906 in miR-133a1 were associated with the risk of asthma in a Chinese Han population [46]. Paula *et al* analysed the association of rs13040413 and rs200375711 in miR-133a2 with long QT syndrome and observed no association between the two SNPs with long QT syndrome [47]. In the present study, we investigated the association of rs6062251 in the near Gene-3 of miR-133a2 with the initiation and development of cervical cancer. These results showed that the genotypic frequencies were significantly different between control, CIN and cervical cancer groups. The inheritance analysis showed that T/T-C/C genotype were correlated with increased risk of progression from CIN to cervical cancer in the overdominant model (*P*=0.007, OR=1.45; 95%CI: 1.11-1.90).

In recent years, miR-100 has been reported to target numerous biomolecules associated with carcinogenesis, suggesting that miR-100 functions as both a tumour promoter and a tumour suppressor. In cervical cancer, miR-100 functions as a potential tumour suppressive miRNA involved in tumour occurrence, development and drug resistance [48]. Li *et al*. [49] reported the reduced expression of miR-100 in CIN and cervical cancer. Zhu *et al*. reported that a SNP in miR-100 was associated with various types of diseases (Table 7) [50]. In the present study, we did not observe any association of rs1834306 in pri-miR-100 with the initiation and development of cervical cancer in a Chinese Han population. Similarly, Xiong *et al*. detected no relationship between rs1834306 in pri-miR-100 and cervical cancer risk in a Chinese Han population (Table 7) [51].

In current study, our results showed the allele or genotype of given miRNA played different roles in normal tissue, CIN and/or cervical cancer. The reason for the discrepancies could be the onset of cervical cancer is a multistep process that means progression of cervical cancer is from normal tissue to invasive cervical cancer through a series increasing grades of squamous intraepithelial lesions [52]. Among this multistep process, the miRNAs expression and their targets have shown discrepancies. For example, the expression of miR-145, miR-218 and miR-100 was progressive reduced and down-regulated from CIN 1 to 3 and cervical cancer versus normal tissue [53–55]. Furthermore, the miRNA expression alternation could change their targets function. In 2012, Qin *et al*. reported that miR-133b expression in CIN 2, 3 and cervical carcinoma had a gradual raise, which increased AKT and MAPKs (ERK1 and ERK2) phosphorylation augmenting tumorigenesis [56]. Their finding indicated miRNAs could play different roles in the progression of cervical cancer. Therefore, the different alleles or genotypes of SNPs in these miRNAs could function differently in the multistep stages of cervical cancer by altering their expression level and the binding affinity between them and their targets.

In summary, we investigated the association of the SNPs in five anti-oncogene miRNAs (pri-miR-218-2, pri-miR-145, pri-miR-133a2, pri-miR124-1 and pri-miR-100) with the CIN and cervical cancer in a Chinese Han population. The results showed that the A allele of rs11134527 in pri-miR-218-2 maybe associated with decreased risk of cervical cancer (OR=0.836; 95%CI: 0.708~0.988) and may be a protective factor for the initiation of CIN (OR=0.786; 95%CI: 0.653~0.946), and the C allele of rs531564 in pri-miR-124-1 may be associated with increased risk of cervical cancer (OR=1.341; 95%CI: 1.054-1.706) and maybe a risk factor for the progress form CIN to cervical cancer (OR=1.385; 95%CI: 1.054-1.819) in a Chinese Han population.

## MATERIALS AND METHODS

### Ethics statement

The present study was approved through the Institutional Review Board of the 3rd Affiliated Hospital of Kunming Medical University. The methods of this investigation were performed in accordance with the approved guidelines and principles expressed in the Helsinki Declaration of 1975, which was revised in 2008. All participants provided written informed consent.

### Subjects

A total of 400 CIN and 609 cervical cancer patients were recruited at the 3rd Affiliated Hospital of Kunming Medical University from 2011 to 2015. The diagnosis of CIN and cervical cancer was confirmed by pathological examination according to “Diagnosis and Treatment Obstetrics and Gynaecology” and FIGO stage (International Federation of Gynaecology and Obstetrics, 2009). Subjects with oncotherapy history or other malignancy or incomplete clinical data were excluded from the present study. During the same period, 583 healthy women undergoing routine health check-ups at the same hospital were designated as the control group. The inclusion criteria of control subjects were HPV-negative women without any cervical lesions. All participants were self-reported as ethnically Han.

### Genomic DNA extracted

Genomic DNA was extracted from peripheral lymphocytes using the QIAamp Blood Mini Kit (Qiagen, Hilden, German).

### MicroRNA selection and sequencing

A total of nineteen miRNAs associated with cervical cancer [18] were sequenced (the sequencing ranged from 200bp upstream to 200bp downstream of miRNA genes) in 50 healthy individuals to search for SNPs (miR-124-1, miR-9-2, miR-100, miR-101-2, miR-214, miR-218-1, miR-218-2, miR-424, miR-7-1, miR-7-2, miR-7-3, miR-21, miR-23B, miR-31, miR-127, miR-133a2, miR-145, miR-205 and miR-3529).

### SNP genotyping using TaqMan assay method

SNPs, namely, rs11134527 in pri-miR-218-2, rs74693964 in pri-miR145, rs6062251 in pri-miR-133a2, rs531564 in pri-miR-124-1 and rs1834306 in pri-miR-100, were genotyped through PCR amplification using the TaqMan assay. The TaqMan assays including the primers and probes were designed and ordered by Applied Biosystems (https://bioinfo.appliedbiosystems.com/genome-database/snp-genotyping.html). Briefly, these SNPs were genotyped in 384-well plates using TaqMan assays in the QuantStudio 6 Flex Fast Real-Time PCR System. Amplification was performed in a 5-μl reaction volume consisting of 2.5 μl 2 × TaqMan Master Mix, 0.125 μl 40 × Primer and TaqMan Probe (FAM VIC) dye mix, 1.375 μl ddH_2_O, and 1 μl template DNA (substituted by equivalent ddH_2_O in negative control). The following PCR cycle conditions were used: 95°C for 10 min; PCR stage 92°C for 10 s and 60°C for 1 min repeated in 40 cycles. Data acquisitions and analysis were performed on QuantStudio™ real-time PCR software. To identify the accuracy of SNP genotyping using the TaqMan assay, three positive controls and one negative control were simultaneously genotyped using the TaqMan assay.

### Statistical analysis

The allelic and genotypic frequencies of the SNPs were calculated using a direct-counting method. Hardy-Weinberg equilibrium (HWE) was tested for the SNPs in the control group. A *χ^2^* test was used to determine differences in allelic and genotypic frequencies, and the odds ratios (OR) with associated 95% confidence intervals (CIs) of allele and genotype-specific risks were calculated. The ages among different groups were compared using one-way ANOVA, followed by the LSD test for multiple comparisons correction. The association of each SNP with CIN and cervical cancer was analysed for the model of inheritance using SNPStats software [57]. The Akaike information criterion (AIC) and Bayesian information criterion (BIC) were used to determine the best-fit model for each SNP. The model with the smallest AIC and BIC values corresponds to minimal expected entropy. The statistical analyses were performed using SPSS 13 (Chicago, IL). A *P* value less than 0.05 was considered statistically significant.

## Abbreviations

SNP: single nucleotide polymorphisms
miRNA: microRNA
CIN: cervical intraepithelial neoplasia
HPV: human papillomavirus
HWE: Hardy-Weinberg equilibrium
OR: odds ratios
CI: confidence intervals
AIC: Akaike information criterion
BIC: Bayesian information criterion.
